# ICP5249 Promotes Hair Growth by Activating the AMPK-Autophagy Signaling Pathway

**DOI:** 10.4014/jmb.2406.06015

**Published:** 2024-07-26

**Authors:** Jung Ok Lee, Yu-jin Kim, You Na Jang, Jung min Lee, Kayoung Shin, Sekyoo Jeong, Hwa-Jee Chung, Beom Joon Kim

**Affiliations:** 1 Department of Dermatology, College of Medicine, Chung-Ang University, Seoul 06974, Republic of Korea; 2 Department of Medicine, Graduate School, Chung-Ang University, Seoul 06973, Republic of Korea; 3 R&D Center, Incospharm Corporation, Daejeon 34000, Republic of Korea

**Keywords:** Human dermal papilla cells (hDPCs), hair growth, autophagy, AMPKα, ICP5249

## Abstract

Autophagy is essential for regulating hair growth. Accordingly, we developed autophagy activator ICP5249 (pentasodium tetracarboxymethyl palmitoyl dipeptide) and investigated its potential role in hair growth. We evaluated its effect on hair growth using *in vitro* human dermal papilla cells (hDPCs) culture model, human hair follicles (hHFs) organ culture model, and telogenic mouse model. ICP5249 increased hDPCs proliferation and alkaline phosphatase (ALP) expression. It also increased microtubule-associated protein (MAP) light chain 3-II (LC3-II) expression and AMP-activated protein kinase α (AMPKα) and unc-51-like kinase 1 (ULK1) phosphorylation in hDPCs. ICP5249 extended the length of hHFs and increased LC3-II please revised from LC3 II to LC3-II in all manuscript expression. Consistently, ICP5249 also significantly increased hair growth area, dermis thickness, and anagen and telogen ratio in telogenic mice. Furthermore, it upregulated Ki-67 and LC3-II expression and AMPKα phosphorylation on the mice’s dorsal skin. To investigate whether AMPK regulates ICP5249-induced hair growth, following treatment with the compound C, AMPK inhibitor, the activity of ICP5249 was evaluated.

The effects of ICP5249 on hair growth were assessed following pretreatment with the AMPK inhibitor compound C. The results showed that compound C suppressed ICP5249-mediated proliferation and hair inductivity in hDPCs. Additionally, compound C inhibited ICP5249-mediated hair growth area, dermis thickness, anagen and telogen ration, and LC3-II expression in mice, suggesting that ICP5249 promotes hair growth by modulating autophagy, with AMPKα playing a regulatory role in this process. Taken together, we demonstrate that ICP5249 has the potential as an ingredient for improving hair growth.

## Introduction

There are a wide variety of causes for hair loss, such as heredity, hormone imbalance, stress, malnutrition, aging, and certain environmental conditions [1]. Hair loss affects an individual’s social interactions and physical appearance. Currently, there are two Food and Drug Administration (FDA)-approved drugs for hair loss treatment: minoxidil (MNX) and finasteride [2, 3]. However, the efficacy of MNX and finasteride is limited in advanced hair loss. Additionally, their long-term use is associated with side effects, including infertility and vasodilatory edema [4, 5]. Therefore, developing safe hair loss therapy with fewer side effects is necessary.

The hair cycle is dynamic and cyclic, encompassing three phases: anagen (growth), catagen (regression), and telogen (rest). The transition from telogen to anagen is essential for initiating new hair growth [6]. Human dermal papilla cells (hDPCs) play a crucial role in regulating hair cycle, especially in initiating and sustaining the anagen phase [7, 8]. The regulation of these phases is controlled by intricate cellular mechanisms, including proliferation and apoptosis [9]. The anagen of hDPCs starts with the main cell signaling pathways, including Wnt, sonic hedgehog (Shh), fibroblast growth factor (FGF), Notch, and bone morphogenetic protein (BMP) pathways [10]. The Wnt pathway is the primary regulator of morphogenesis initiation in human hair follicles (hHFs). Interruption of β-catenin signaling in the hDPCs induces catagen and inhibits anagen [11, 12]. BMP is a key member of the TGF-β superfamily. The expression of BMP-2 and BMP-4 fluctuates periodically throughout the hair cycle [13].

The Notch signaling pathway is critical for preserving HF structure and promoting HF development and re-epithelialization [14]. The typical characteristics of hHFs entering catagen are the stop of the hair shaft growth, decline in hDPCs proliferation and differentiation, initiation of apoptosis, and rapid degeneration of hHFs. Epithelial cells of the hair matrix and outer root sheath (ORS) undergo apoptosis, and human dermal papilla (hDP) volume becomes smaller [15]. The degeneration of hHFs is highly regulated during catagen, and many keratinocytes in hHFs undergo apoptosis [16].

Autophagy is intracellular degradation during which cytoplasmic substances are transmitted to lysosomes. Besides its critical role in degrading and recycling damaged organelles to maintain cellular homeostasis, it plays various roles in cellular pathological processes [17, 18]. Autophagy is extensively involved in skin homeostasis; hence, defects in autophagy can influence skin aging [19, 20]. In skin homeostasis, autophagy targets various organelles and proteostasis pathways, including nucleus and mitophagy elimination during keratinocyte differentiation [21, 22], anti-inflammatory processes [23], and melanogenesis and antioxidant activity in melanocytes [24].

According to a previous report, autophagy inhibitor 3-MA induces the early start of catagen and prolongs telogen, whereas induction of autophagy by mTOR inhibitor rapamycin promotes hair growth [25]. A more active autophagic flux occurs in hair matrix keratinocytes during anagen compared with catagen, indicating that autophagy is involved in extending the growth phase of hair [26]. Furthermore, genetic mutation of autophagy in hHFs causes premature catagen and heightened apoptosis of matrix keratinocytes, highlighting the significance of autophagy for maintaining hHFs growth.

AMP-activated protein kinase (AMPK), a highly conserved serine/threonine-protein kinase throughout evolution, serves as a cellular energy sensor that enhances catabolism and inhibits anabolism [27, 28]. AMPKα phosphorylation at Thr^172^ is required for maximum AMPK activity [29]. Additionally, AMPKα regulates autophagy via direct Unc-51-like kinase-1 (ULK-1) phosphorylation. Furthermore, the AMPK/ULK1 pathway is involved in regulating autophagy. Inhibition of this pathway can reduce autophagosome production and LC3 expression [30, 31].

ICP5249 is an autophagy-modulating peptide derivative (AMPedTM) developed by Incospharm Co., Ltd. In the present study, we examined the impacts of ICP5249 on hair growth using *in vitro*, ex vivo, and in vivo models. Additionally, we analyzed the mechanism by which ICP5249 promotes hair growth. We discovered that the effectiveness of ICP5249 in enhancing hair growth is attributed to its promotion of autophagy, which is regulated by AMPK activation.

Finally, this study suggests that ICP5249 is a novel substance that could contribute to improving hair health.

## Materials and Methods

### ICP5249 and Cell Culture

Autophagy inducer ICP5249, *i.e.*, 2-[2,6-bis-(bis-carboxymethyl-amino)-hexanoylamino]-6-hexadecanoylamino-hexanoic acid, was created through solid-phase peptide synthesis with fluorenylmethyloxycarbonyl chloride (Fmoc-Cl) and purified using reverse-phase high-performance liquid chromatography (RP-HPLC) (Incospharm Corp., Republic of Korea). hDPCs were obtained from PromoCell (Germany) and cultured in follicle DP cell growth medium (PromoCell) in a humidified incubator at 37°C with 5% CO_2_.

### Reagents and Antibodies

MNX and compound C were purchased from Sigma-Aldrich (USA). LC 3B I/II was purchased from Abcam (UK). Antibodies for p-AMPKα (Thr^172^), AMPKα, p-ULK1 (Ser^555^), ULK1, and β-actin were purchased from Cell Signaling Technology (USA). Ki-67 and alkaline phosphatase (ALP) were purchased from Invitrogen (USA).

### Proliferation of hDPCs

hDPCs were seeded in 96-well plates (Corning Inc., USA). Then, the cells were treated with 0, 0.625, 1.25, 2.5, 5, or 10 μg/ml ICP5249 for 24 h. The cells were pretreated with 1 μM compound C prior to adding ICP5249 to inhibit AMPKα. After 24 h, cell viability was quantified using a water-soluble tetrazolium salt (WST-8) assay kit (QuantiMax, BIOMAX). Absorbance was measured at 450 nm using a SpectraMax i3x microplate reader (Molecular Devices).

### Reverse-Transcription and Quantitative PCR

Total RNA was extracted using TRIzol Reagent (Invitrogen, USA). Single-strand complementary DNA (cDNA) was synthesized through reverse transcription using PrimeScript TM RT Master Mix (Takara, Japan). The resulting cDNA was subjected to a quantitative polymerase chain reaction (qPCR) on a CFX96 thermocycler (Bio-Rad, USA) using qPCR PreMIX SYBR Green (Enzynomics, Republic of Korea). Gene expression levels were calculated as a cycle threshold (Ct) value using the 2^−ΔΔCT^ quantification method and normalized to that of glyceraldehyde-3-phosphate dehydrogenase (*GAPDH*). Table 1 lists the primers used for qPCR.

### Western Blot Analysis

Protein quantification was performed using a Pierce BCA Protein Assay Kit (Thermo Fisher Scientific). Protein samples were run on a 10% SDS‐PAGE gel and transferred to a PVDF membrane. After blocking with 5% skim milk in Tris-buffered saline (TBS) containing 0.1% Tween-20 (TBS-T) for 1 h, the membrane was incubated with primary antibodies overnight at 4°C, washed, and incubated with horseradish peroxidase–conjugated anti-mouse (Vector Laboratories Inc., USA) or anti-rabbit (Vector Laboratories Inc.) secondary antibodies. Immunodetection was performed using an Amersham ECL kit (GE Healthcare, USA) according to the manufacturer’s protocol. Protein bands were visualized using a ChemiDoc MP Imaging System (Bio-Rad Laboratories, Inc.) and analyzed using the NIH Image J software (USA).

### Spheroid Formation and Hanging Drop Culture

Three-dimensional (3D) cultures of hDPCs were performed for spheroid generation. First, cells (1 × 10^5^ cells/ml) were dispensed (30 μl/well) into clear 96-well round-bottom ultra-low attachment microplates (Nexcelom Bioscience, USA). Then, the spheroids were treated with ICP5249 for 6 h. Spheroid diameters were measured using phase-contrast microscopy (Leica, Germany).

### Immunocytochemistry

The cells were fixed with 4% paraformaldehyde (PFA) for 30 min, washed with phosphate-buffered saline (PBS), blocked with 3% bovine serum albumin (BSA) and 0.2% Triton X-100 in PBS at room temperature (RT) for 1 h, and incubated overnight at 4°C with primary antibodies. After washing with PBS, the cells were incubated with anti-rabbit IgG-FITC secondary antibodies (Santa Cruz Biotechnology, USA) for 1 h in the dark. The nuclei were counter stained with 4’,6-diamidino-2-phenylindole (DAPI; Immuno Bioscience Corp., USA). Afterward, the cells were observed using confocal microscopy (LSM 800, Carl Zeiss, Germany).

### hHF Organ Culture

hHFs were obtained from the Dankook University Hospital (IRB approval number; DKUH 2021-12-025), alongside written informed consent from respective patients. The isolated anagen hHFs were cultured in 500 μl of Williams E medium (Gibco, USA) at 37°C with 5% CO_2_. After 24 h, the hHFs were cultured with ICP5249 (0, 0.5, 1.25, or 2.5 μg/ml) for 4 days. hHFs images were obtained using a stereo microscope (Zeiss). hHFs elongation was analyzed using ImageJ (version 1.52a).

### Histology and Immunohistochemistry

Dorsal skin tissues from each mouse were fixed with 10% formalin, embedded in paraffin, and cut into sections, which were subsequently stained with hematoxylin and eosin (H&E). For immunohistochemistry, the sections were incubated with primary antibodies. The stained slides were photographed using a slide scanner (Pannoramic MIDI; 3DHISTECH Ltd., Hungary) and observed using Case Viewer software. The number of HFs was counted on a cropped image within a fixed area (1 × 1 mm).

### Immunofluorescence

The cells and paraffin-embedded section tissues were fixed with 4% paraformaldehyde (PFA) for 30 min, blocked with 3% BSA and 0.2% Triton X-100 in PBS, and incubated with primary antibodies overnight at 4°C. After washing, the cells were incubated with anti-rabbit IgG-FITC secondary antibodies (sc2359; Santa Cruz Biotechnology) for 1 h. The nuclei were stained with DAPI (4',6-diamidino-2-phenylindole; Immuno Bioscience Corp., USA). Then, the cells were observed using confocal microscopy (LSM 880, Zeiss).

### Hair Regeneration Model

Six-week-old female C57BL/6 mice were obtained from Saeron Bio Inc. and acclimated for one week with freely accessible food and water. Telogen-anagen transition mice were prepared by shaving the dorsal skin during the telogen phase [24]. The mice were randomly divided into four groups: normal control, 3% MNX, ICP5249 (250 mg/kg), and ICP5249 (500 mg/kg). ICP5249 was administered orally, while MNX was applied topically 5 times a week for 2 weeks. Compound C (10 mg/kg-body-weight) was intraperitoneally injected 1 h before ICP5249. Control animals received a similar volume of dimethyl sulfoxide (DMSO). The Institutional Animal Care and Use Committee of Chung-Ang University approved all animal experiments, which were performed according to the laboratory animal care principles set by the National Institutes of Health (NIH).

### Statistical Analysis

The results were expressed as the mean ± standard deviation (SD) of at least 3 independent experiments. The data were analyzed using a one-way analysis of variance (ANOVA), followed by a Bonferroni post hoc test. All statistical analyses were performed using the GraphPad Prism 7.0 (GraphPad Software Inc., USA). Differences with *p* < 0.05 denoted statistical significance, as indicated on graphs using the following symbols: *, *p* < 0.05; **, *p* < 0.01; ***, *p* < 0.001; and ****, *p* < 0.0001.

## Results

### ICP5249 Promotes the Proliferation and Hair Inductivity of hDPCs

**Fig. 1:**
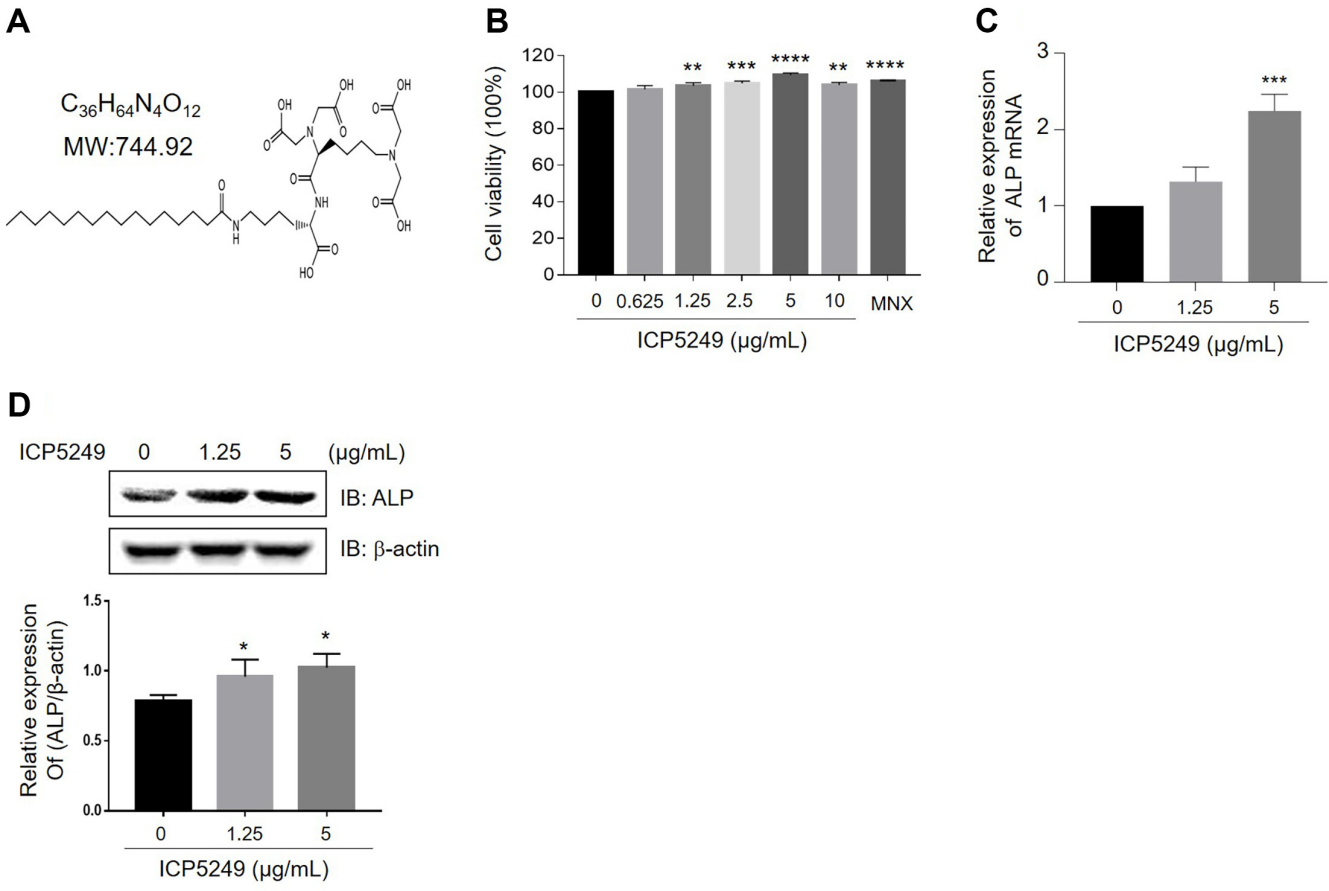
ICP5249 increases the proliferation of hDPCs. (**A**) The structure of ICP5249. (**B**) The viability of hDPCs was assessed after treatment with ICP5249 (0, 0.625, 1.25, 2.5, 5, and 10 μg/ml) for 24 h using a WST-8 assay. (**C**) hDPCs were treated with ICP5249 (0, 1.25, and 5 μg/ml; 12 h), and ALP mRNA expression was analyzed using qPCR. (**D**) hDPCs were treated with ICP5249 (0, 1.25, and 5 μg/ml; 24 h), and ALP protein expression was analyzed using western blotting. *, *p* < 0.05; **, *p* < 0.01; ***, *p* < 0.001; ****, *p* < 0.0001 compared to the control group.

Fig. 1A presents the structure of ICP5249. ICP5249 increased hDPC proliferation by 5%, 7%, and 12% at 2. 5, 5, and 10 μg/ml, respectively, with MNX as a positive control (Fig. 1B). ALP improves the hair-inductive capacity of hDPCs [25, 26]. The levels of ALP mRNA and protein increased in a manner that depended on the dose (Fig. 1C and 1D).

### ICP5249 Activates Autophagy

**Fig. 2:**
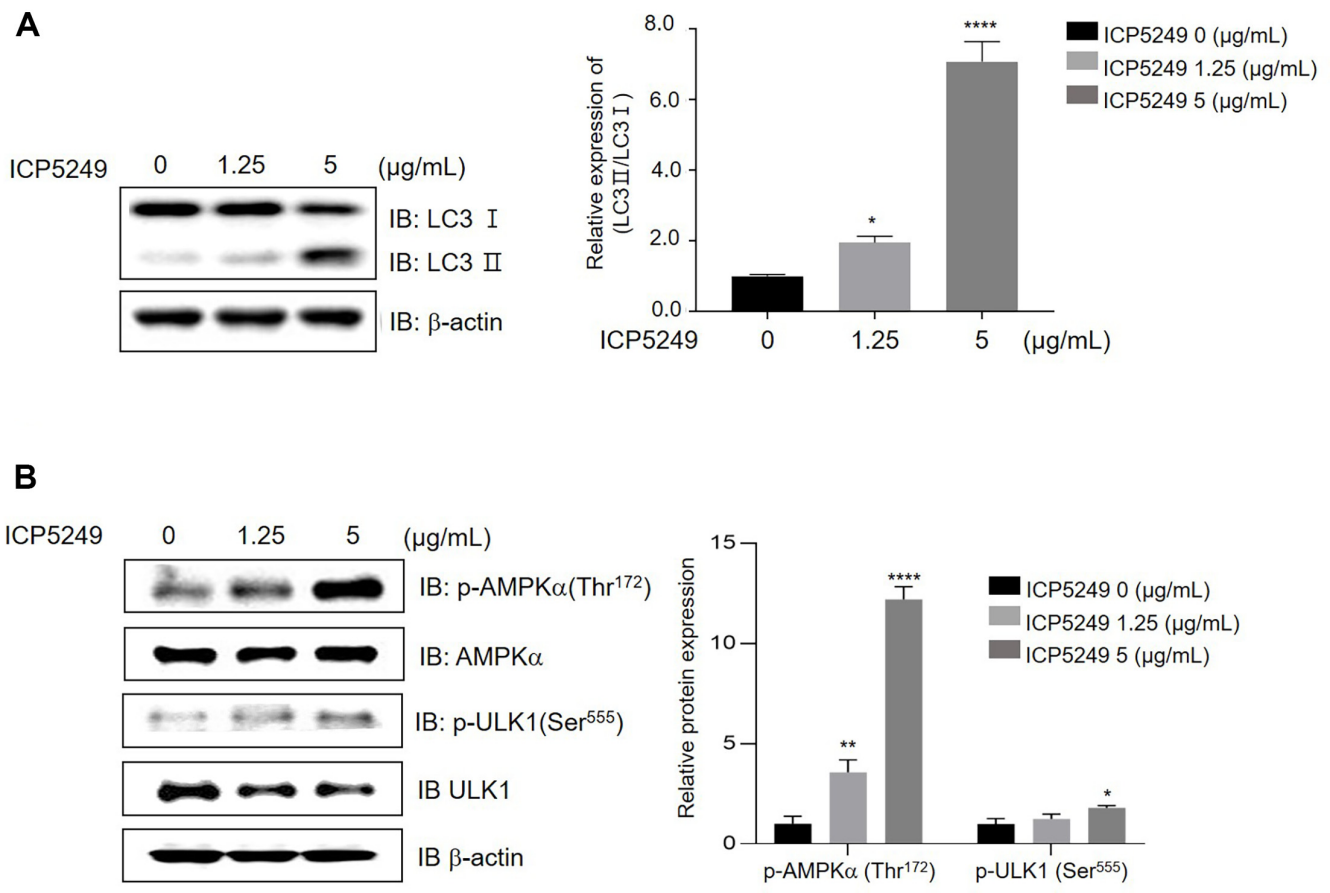
ICP5249 activates autophagy in hDPCs. (**A**) hDPCs were treated with ICP5249 (0, 1.25, and 5 μg/ml; 24 h), and protein expression of LC3-I/II and β-actin was assessed using western blotting. (**B**) hDPCs were treated with ICP5249 (0, 1.25, and 5 μg/ml; 1 h), and protein expression of p-AMPKα, p-ULK1, AMPKα, ULK1, and β-actin was examined using western blotting.

The conversion of LC3-I (free form) to LC3-II (lipid-conjugated form) is a significant step in autophagosome formation, with the LC3-II amount correlating with autophagosome quantity [27, 28]. ICP5249 dose-dependently elevated LC3-II expression, suggesting activation of autophagy in hDPCs (Fig. 2A). ICP5249 increased LC3-II expression in a dose-dependent manner, indicating that ICP5249 activates autophagy in hDPCs (Fig. 2A). Moreover, ICP5249 dose-dependently enhanced the phosphorylation of AMPKα (Thr^172^) and ULK1 (Ser^555^) (Fig. 2B).

### ICP5249 Activates Autophagy by the AMPK Pathway

**Fig. 3:**
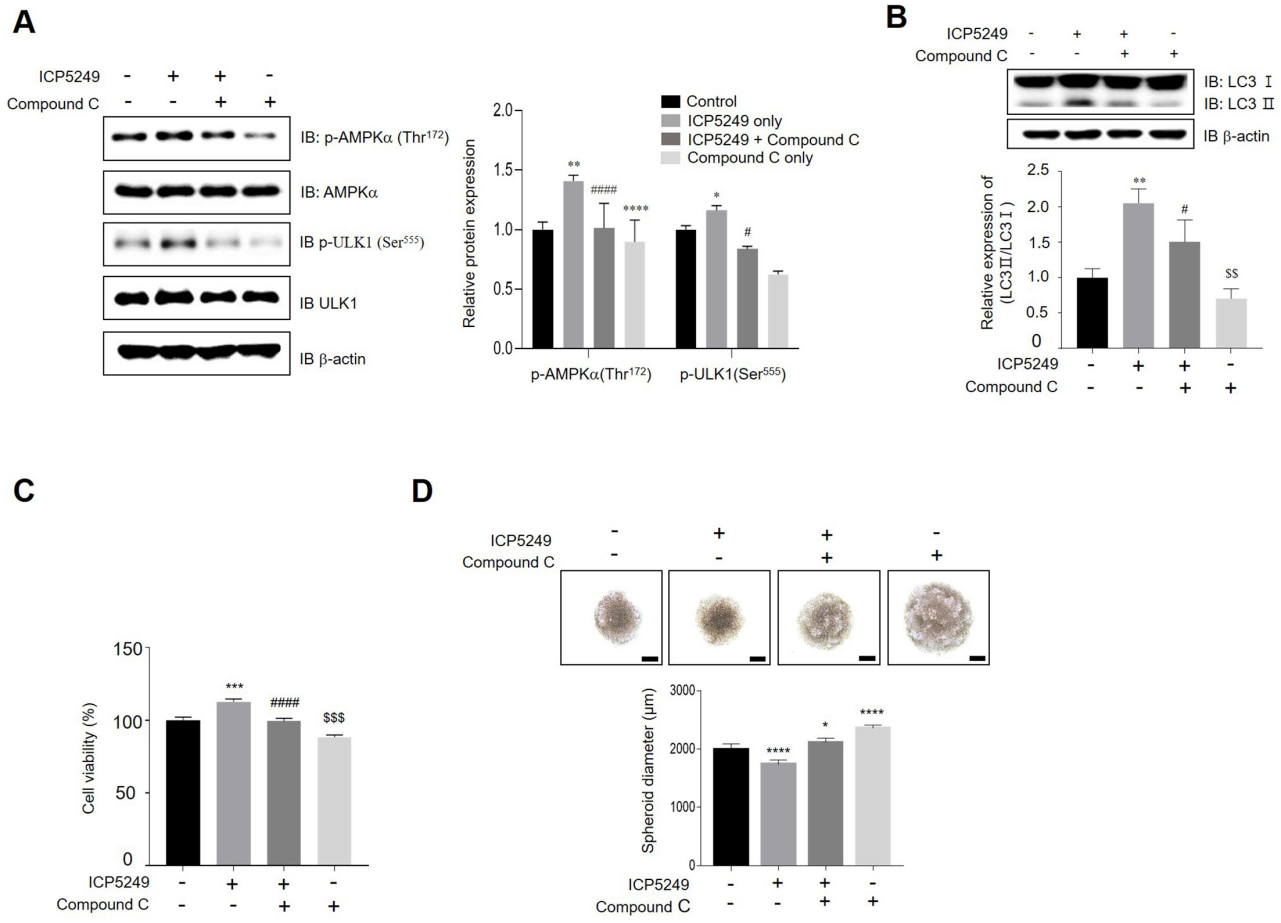
ICP5249 increases the proliferation and inductivity of hDPCs through AMPK-mediated autophagy. (**A**) hDPCs were pretreated with compound C for 30 min and then treated with ICP5249 (5 μg/ml) for 1 h. Protein expression of p-AMPKα, p-ULK1, AMPKα, ULK1, and β-actin was assessed using western blotting. (**B**) hDPCs were pretreated with compound C for 30 min and then treated with ICP5249 (5 μg/ml) for 24 h. Protein expression of LC3-I/II and β-actin was assessed using western blotting. (**C**) Cell viability was measured using a WST-8 assay after pretreatment with compound C for 30 min, followed by treatment with ICP5249 (5 μg/ml) for 24 h. (**D**) 3D-spheroid formation by hDPCs treated with vehicle, ICP5249 (5 μg/ml), ICP5249 in combination with compound C, or compound C for 24 h. Spheroid diameters were quantified. The results are expressed as the mean ± SD. *, *p* < 0.05; **, *p* < 0.01; ***, *p* < 0.001; ****, *p* < 0.0001 compared with the control group.

Pretreatment with compound C suppressed ICP5249-mediated phosphorylation of AMPKα and ULK1 (Fig. 3A). Additionally, AMPKα inhibition attenuated LC3-I and LC3-II expression, suggesting that AMPKα is an upstream regulator of ICP5249-induced autophagy (Fig. 3B). Moreover, pretreatment with compound C inhibited the ICP5249-induced increase in hDPC proliferation (Fig. 3C). We performed a hanging drop array (HDA) technique to examine the effect of ICP5249 on hair inductivity in hDPCs. Fig. 3D shows that ICP5249 promotes spheroid aggregation compared to control, and these effects were inhibited by compound C pretreatment. Thus, ICP5249-mediated proliferation and hair inductivity of hDPCs are regulated by the AMPKα-autophagy signaling pathway.

### ICP5249 Increases the Length of hHFs

**Fig. 4:**
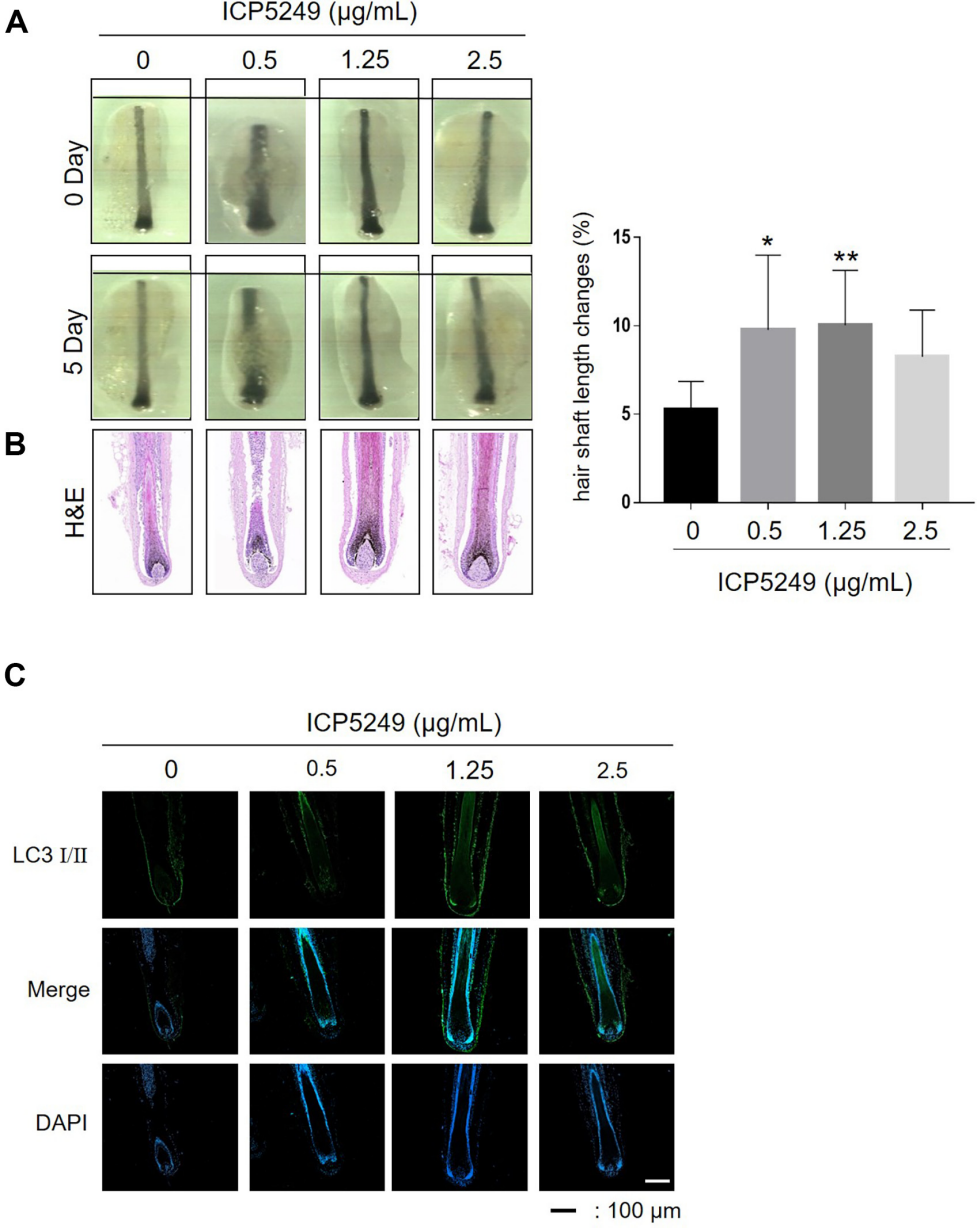
ICP5249 enhances human hair shaft growth. hHFs were treated with ICP5249 (0, 0.5, 1.25, or 2.5 μg/ml) for 5 days. (**A**) The length of hHFs was analyzed under a stereomicroscope on days 0 and 5. ImageJ software was used to quantify each hair shaft’s relative length. (**B**) H&E staining and (**C**) immunofluorescence analysis of LC I/II expression in hHFs treated with ICP5249. The results are expressed as the mean ± SD. **, *p* < 0.01; ***, *p* < 0.001; ****, *p* < 0.0001 compared with the control group.

ICP5249 significantly enhanced hair shaft growth. The maximum effect was observed at concentrations of 0.5 and 1.25 μg/ml, resulting in a hair shaft increase of more than 10% compared to the control (Fig. 4A). To further analyze these effects, we examined control and ICP5249-treated (1.25 μg/ml) samples, focusing on the hair bulb area (encompassing the hair bulb/DP), using H&E staining after 5 days in the hHFs culture. The hair bulbs and suprabulbar region of hHFs treated with 1.25 μg/ml ICP5249 exhibited a sustained and statistically significant enlargement compared to vehicle-treated hHFs (Fig. 4B). Additionally, LC3-I/II expression increased in the ICP5249-treated group compared to the control group (Fig. 4C).

### ICP5249 Accelerates Hair Growth by AMPKα-Mediated Autophagy in C57BL/6 Mice

**Fig. 5:**
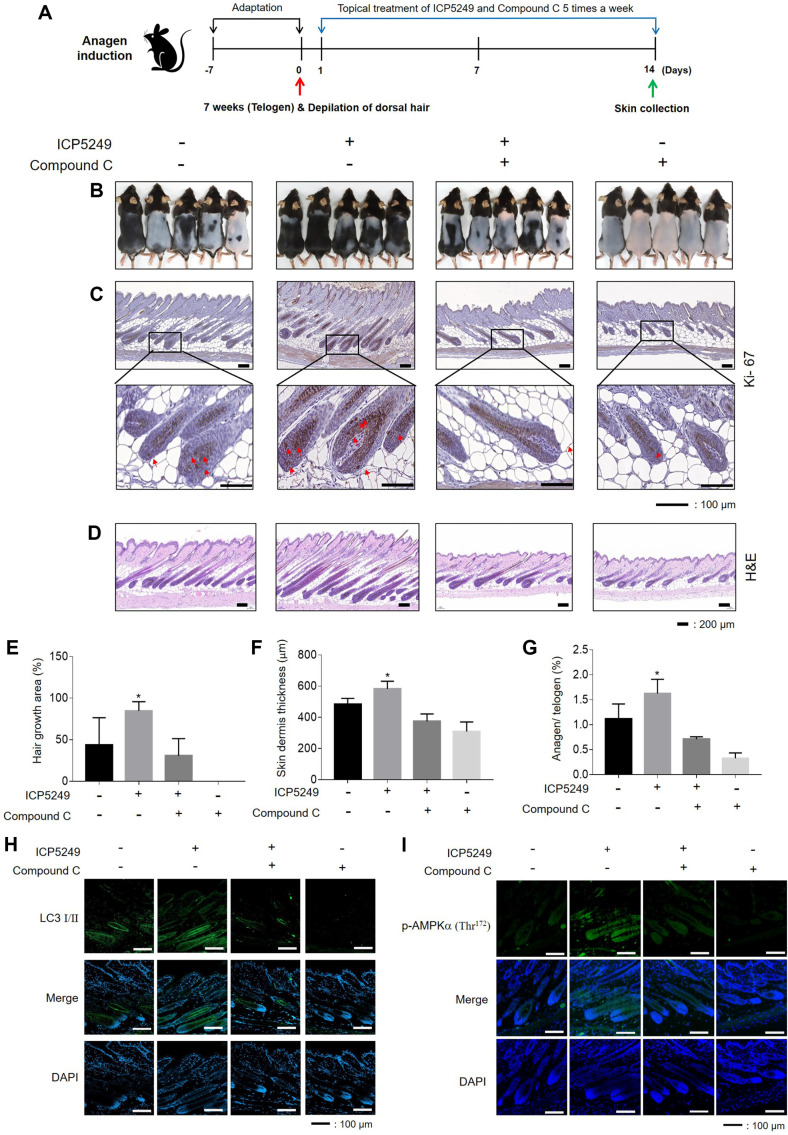
ICP5249 increases hair growth through AMPK-mediated autophagy in C57BL/6 mice. The vehicle, ICP5249, ICP5249 in combination with compound C, or only compound C (*n* = 5 per group) was orally administrated 5 times per week for 14 days. (**A**) Experimental treatment and sample collection timetable. (**B** and **E**) Representative photographs and the hair growth area of the back skin for 14 days. (**C–D**) Representative immunohistochemistry images of Ki67 and H&Estained longitudinal sections of skin tissues from mice receiving the indicated treatments on day 14. Scale bar, 200 μm. (**F**) Hair dermis thickness and (**G**) anagen/telogen ratios on day 14. (**H–I**) LC I/II and p-AMPKα (Thr^172^) expression on the dorsal skin on day 14. Representative immunofluorescence images are shown. Scale bar, 400 μm. The results are expressed as the mean ± SD. *, *p* < 0.05; **, *p* < 0.01; ***, *p* < 0.001; ****, *p* < 0.0001 compared with the control group.

Seven-week-old mice were subjected to dorsal skin depilation and received an intraperitoneal injection of compound C (10 mg/kg) or a DMSO solution 1 h before ICP5249 (500 mg/kg) to evaluate whether AMPKα is involved in ICP5249-mediated autophagy related to hair growth (Fig. 5A). ICP5249 increased hair growth compared to the vehicle treatment. However, AMPKα inhibition with compound C suppressed ICP5249-mediated hair growth (Fig. 5B and 5E) and decreased Ki67 expression (Fig. 5C). In ICP5249- and MNX-treated mice, there was a significant increase in interfollicular whole skin thickness (*p* < 0.05) (Fig. 5D and 5F). Examination of representative transverse sections (hair growth phase) confirmed that the number of anagen HFs was higher in the ICP5249-treated groups compared to the vehicle group (Fig. 5D and 5G). Furthermore, the expression of LC3 I/II and p-AMPKα (Thr^172^) increased in the mice’s ICP5249-treated dorsal skin (Fig. 5H and 5I). However, compound C pretreatment inhibited these effects. Therefore, ICP5249 promotes hair growth by activating the AMPKα-autophagy signaling pathway.

## Discussion

The current study unveiled an important role of ICP5249 in promoting hair growth by activating autophagy both *in vitro* and in vivo. Specifically, we found that AMPKα is a crucial regulator of ICP5249 to activate autophagy. Compound C, an AMPKα inhibitor, prevented ICP5249-mediated hair growth by inhibiting autophagy.

Autophagy is pivotal in the degradation and recycling of cellular components. Its dysregulation is implicated in hair growth disorders [32]. Min Chai *et al*. reported that quiescent hHFs in the telogen phase can initiate the anagen phase by treatment with autophagy activator, including a-ketoglutarate (α-KG), a-ketobutyrate (a-KB), and metformin [25]. Interestingly, these compounds share the common feature of promoting hair growth by activating autophagy through AMPK. As shown in Fig. 2A and 2B, we observed that ICP5249 increases LC3 II expression and AMPK phosphorylation in hDPCs. Interestingly, AMPK inhibition by compound C prevented ICP5249-mediated hDPCs proliferation and inductivity (Fig. 3C and 3D). Thus, we confirmed that ICP5249 may enhance hair growth by activating the AMPK-autophagy signaling pathway in hDPCs.

LC3 I/II is a key protein involved in the autophagy pathway [33]. LC3I is the cytosolic form of LC3, while LC3-II is the lapidated form associated with autophagosome membranes, playing an essential role in autophagy. The LC3-I to LC3-II ratio is a useful indicator of cellular autophagic activity [34]. A decrease in this ratio usually indicates autophagy upregulation, whereas an increase suggests autophagy downregulation [ 30, 31]. We observed that ICP5249 dose-dependently decreased the LC3-I to LC3-II ratio (Fig. 2A). Consistently, ICP5249 also induced autophagy in hHFs and telogenic mouse model (Fig. 4C and 5H), suggesting that ICP5249 promotes hair growth by inducing autophagy.

Hair follicle stem cells (HFSCs) generally stay quiescent and become active only during the transition from telogen to anagen, ensuring that hHFs enter a new hair cycle [35, 36]. Pingping Sun *et al*. demonstrated that autophagy activates HFSCs by facilitating the shift from HFSC metabolism to glycolysis to supply energy, ultimately initiating the hair follicle cycle and promoting hair growth [37]. Therefore, autophagy is crucial for maintaining a healthy hair cycle. Furthermore, support autophagy activator ICP5249 plays a role in hair health.

Ki-67 is a protein commonly used cellular proliferation marker [38, 39]. It is present in the nuclei of cells during active phases of the cell cycle (G1, S, G2, and M phases), but absent in resting cells (G0 phase). [40, 41] and It is used for assessing proliferation in various tissues and cells, including hHFs [42, 43]. In Fig. 5C, Ki67 expression in the mouse back skin was significantly increased in the ICP5249-treated group. Thus, ICP5249 induces hair growth by increasing hDPCs proliferation.

Ohyama et al. reported that ALP expression serves as a biological marker of hDPCs [44]. The activity of ALP correlates with the hair-inducing capacity of DPCs [45-47]. Hence, ALP is a key player in promoting hair growth. We confirmed that ICP5249 stimulated the expression of ALP mRNA and protein (Fig. 1C–1D). Therefore, ICP5249 positively influences hair growth by increasing hair inductivity. Furthermore, we confirmed that ICP5249-treated hDPCs were more contractile than vehicle-treated hDPCs in the hDPCs hanging drop culture.

Endogenous autophagy was significantly disrupted in early catagen-like miniaturized HFs from the balding scalps of patients with androgenetic alopecia. Furthermore, inhibiting autophagy with 3-MA could induce apoptosis and premature regression of hair follicles, thereby slowing down hair growth in organ-cultured HFs. In summary of the aforementioned findings, dysfunction in autophagy may represent a potential mechanism in androgenetic alopecia [45]. Enhancing autophagy might have positive therapeutic effects in treating male pattern baldness.

ICP5249 is used as a cosmetic raw material and is already registered in the International Cosmetic Ingredient Dictionary (ICID) and the Korean Cosmetic Ingredient Dictionary. Many functional shampoos for hair health are being introduced. It is known that the main function of the shampoos is to remove dead skin cells from the hair scalp to grow healthy hair, or to help nourish and moisturize the scalp to reduce hair loss. ICP5249 demonstrates significant potential as a functional shampoo ingredient due to its ability to increase hair thickness and promote hair growth. Autophagy modulators have emerged as potential therapeutic agents for hair loss, yet their efficacy remains to be fully explored. This study investigates the capacity of autophagy modulators to stimulate hair regeneration, with current data indicating significant effects during the telogen phase. However, their effectiveness in cases of alopecia has not been assessed, underscoring the need for further research into the use of ICP5249 as a potential drug for alopecia treatments.

Taken together, our study suggests that modulating autophagy with ICP5249 could be a promising therapeutic approach for hair loss disorders, such as pattern hair loss and telogen effluvium.

## Supplemental Materials

Supplementary data for this paper are available on-line only at http://jmb.or.kr.
