# Supplementary material for: ICP5249 Promotes Hair Growth by Activating the AMPK-Autophagy Signaling Pathway
Source: J Microbiol Biotechnol. 2024 Jul 26;34(9):1810–8. doi: 10.4014/jmb.2406.06015 (PMC11473489; doi:10.4014/jmb.2406.06015)
Supplement: Supplementary file 1 [file jmb-34-9-1810-supple.pdf]

J. Microbiol. Biotechnol. 2024. 34(9): 1810–1818
https://doi.org/10.4014/jmb.2406.06015

Supplementary Table

Table S1. Primer sequences used for quantification of gene expression.

Gene		Primer sequence (5′→ 3′)
Human ALP	F	ATTGACCACGGGCACCAT
	R	CTCCACCGCCTCATGCA
Human GAPDH	F	TGGAAATCCCATCACCATCTTC
	R	CGCCCCACTTGATTTTGG
